# Effect of chemical mechanical polishing on surface nature of titanium implants FT-IR and wettability data of titanium implants surface after chemical mechanical polishing implementation

**DOI:** 10.1016/j.dib.2016.11.065

**Published:** 2016-11-24

**Authors:** Zeynep Ozdemir, Gul Bahar Basim

**Affiliations:** Ozyegin University, Faculty of Engineering, Mechanical Engineering Department, Nisantepe Mevki, Orman Sokak No: 34-36, Cekmekoy, Istanbul 34794, Turkey

## Abstract

Bioactivity of titanium depends on the quality and characteristics of its surface oxide film. Through implementation of chemical mechanical polishing (CMP) process on titanium plates, a protective oxide (titania) film grows on the titanium based implant surface. In this article, surface properties of the titanium oxide are investigated as a function of the CMP process conditions. Surface responses were evaluated in terms of wettability, nano-scale surface roughness and material removal rates (MRR). Surface chemical compositions were also evaluated through Fourier transform infrared spectroscopy (FT-IR).

**Specifications Table**

**Table:** 

Subject area	*Biomaterials, Surface Characterization*
More specific subject area	*Post CMP surface properties of titanium plates*
Type of data	*Figures*
How data was acquired	*Wettability performance evaluated by KSV ATTENSION Theta Lite Optic Contact Angle Goniometer. The Al*_*2*_*O*_*3*_*based slurry particle size was determined by Beckman Coulter LS 13320 laser light scattering instrument. The surface compositions of the titanium plates were examined by FT-IR spectroscopy by using Attenuated Total Reflectance (ATR) module.*
Data format	*Analyzed*
Experimental factors	*CMP process was conducted by using nano-sized Al*_*2*_*O*_*3*_*particles in pH adjusted slurry both in the absence and presence of the oxidizer (H*_*2*_*O*_*2*_*) to evaluate the nano-scale titania film formation. CMP experiments were conducted by using a Suba IV subpad stacked under a polytex buff pad to smoothen the surface of the metal plates while protecting the micro scale contours. In addition, abrasive papers with various grid sizes were used to reach micro scale roughness.*
Experimental features	*CMP slurry was prepared through ultrasonicating the suspensions long enough by repeated pH adjustment until full stability was achieved. CMP tests were conducted at 70N downforce (equivalent to a 7.88 psi pressure on the 14 by 14 mm sample size).*
Data source location	*Ozyegin University, Faculty of Engineering, Mechanical Engineering Department, Nisantepe Mevki, Orman Sokak No:34-36, Cekmekoy, Istanbul 34794 Turkey*
Data accessibility	*Data is with this article.*

**Value of the data**

• Size distribution and homogeneity of the CMP slurries is important to obtain desired surface properties.
• Wettability and surface roughness evaluations show a correlation to surface structural properties.
• FTIR measurements were conducted to observe the change in surface chemical composition as a function of implemented CMP condition.

## 1 Data

**Fig. 1:**
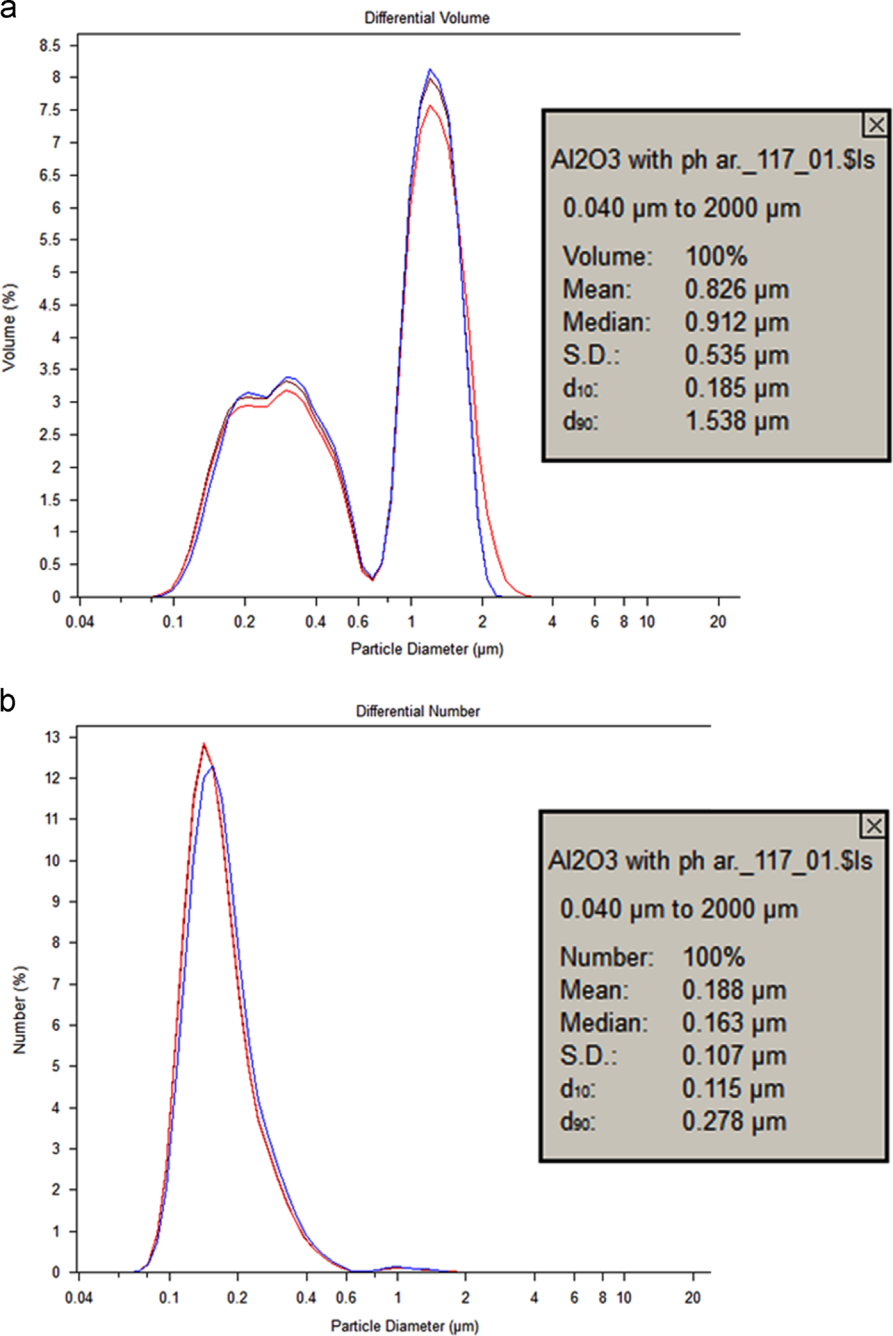
Slurry particle size measurement results (a) volume base and (b) number base distribution.

**Fig. 2:**
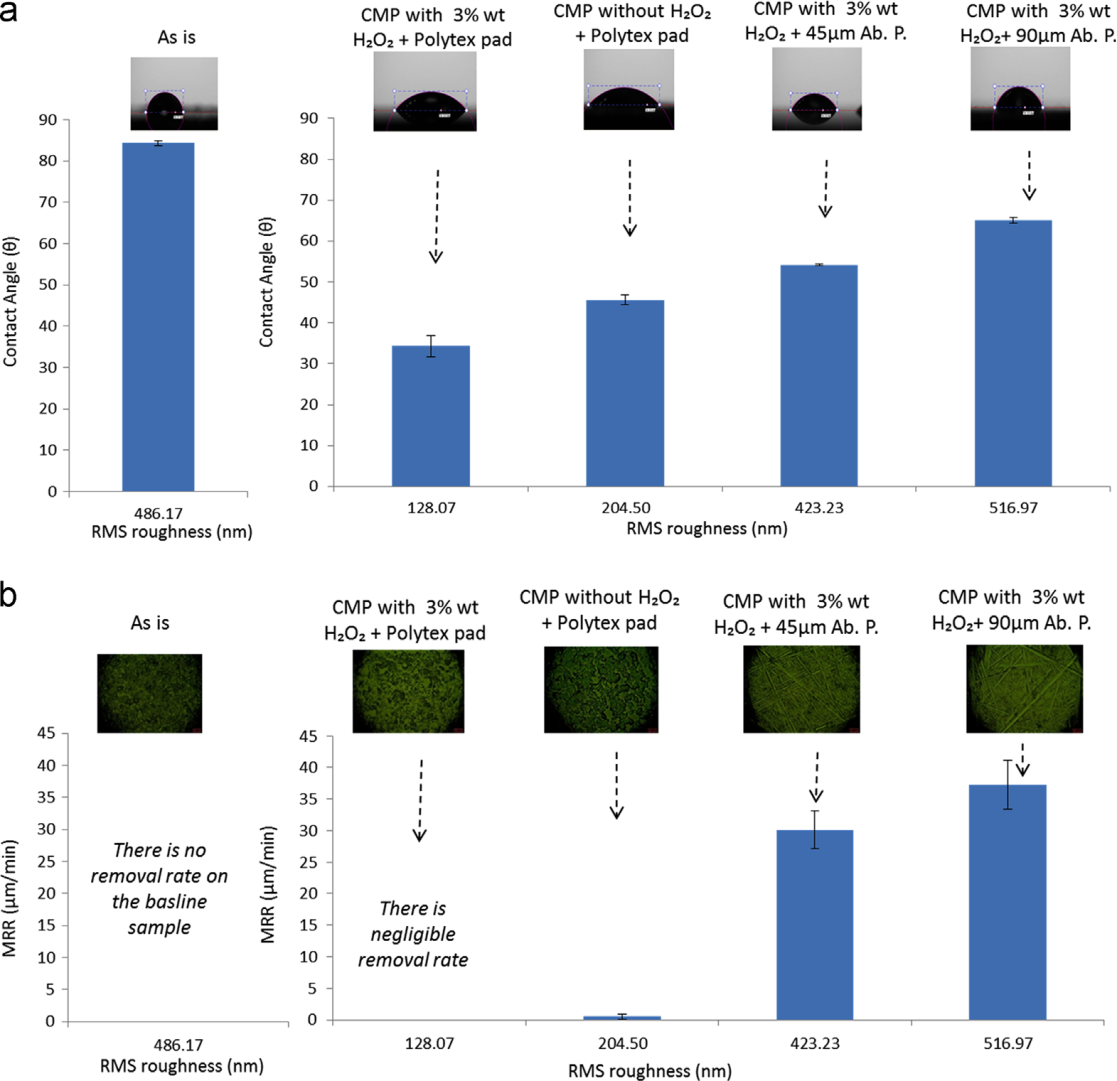
Baseline and CMP treated samples compared for (a) surface wettability performance with contact angle sessile drop images and (b) material removal rate (MRR) response as a function of roughness with optical micrographs at 500X magnification given for the corresponding sample.

**Fig. 3:**
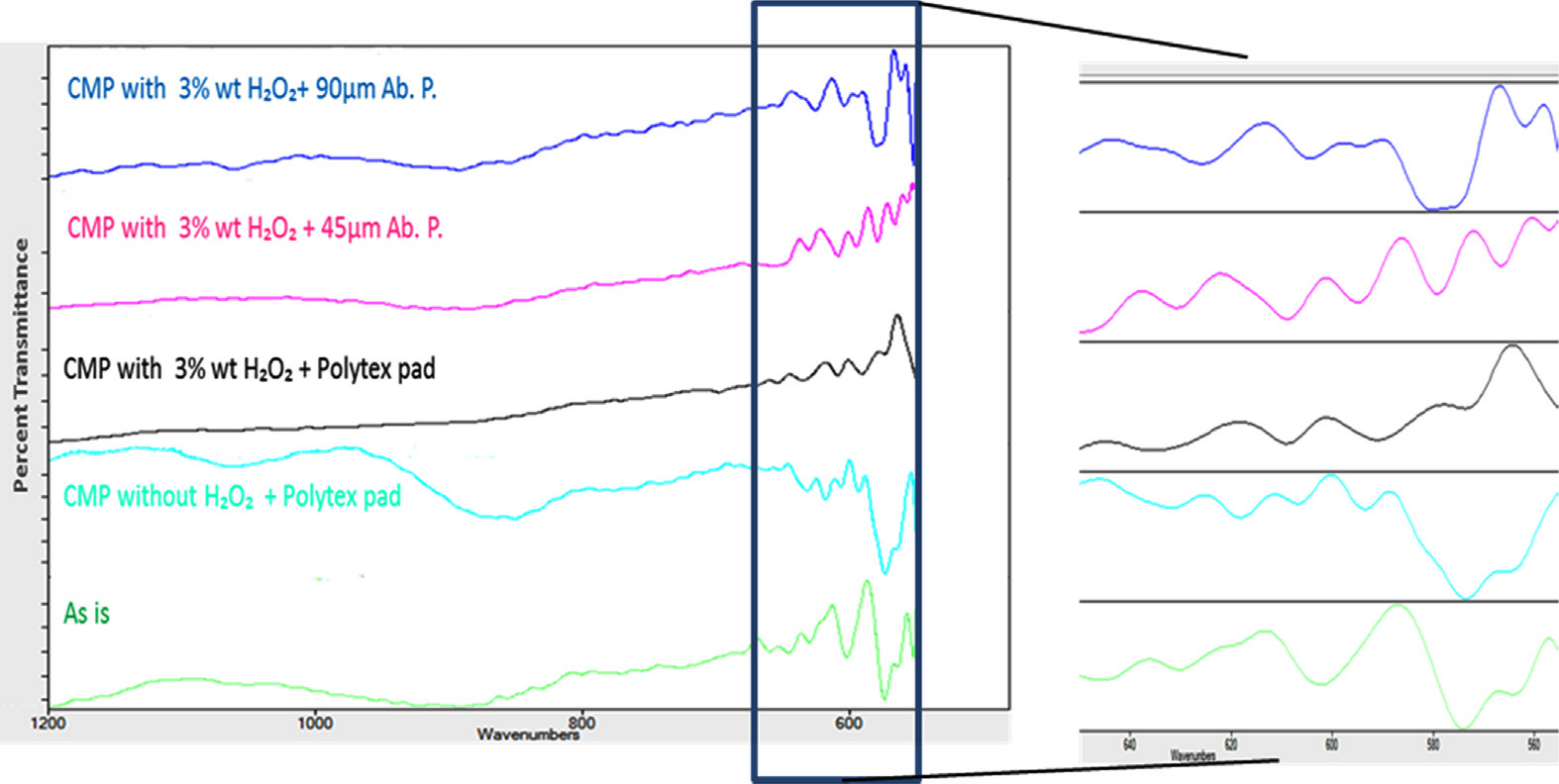
FTIR analyses on the titanium samples as a function of implemented surface treatment.

CMP experiments were performed on the titanium plates by altering the sample surface through various CMP conditions [1]. First, slurry particle size and stability were evaluated through particle light scattering based particle size measurement by using Coulter LS 13 320 equipment as can be seen in Fig. 1. Contact angle measurements (wettability) and root mean square (RMS) surface roughness values were measured by contact angle goniometer and atomic force microscopy (AFM), respectively and summarized in Fig. 2. Furthermore, surface chemical compositions of the titanium samples were studied through FT-IR analyses to observe any chemical changes on the surface layer as seen in Fig. 3.

## 2 Experimental design, materials and methods

Titanium foils with 1mm thickness and 99.6% purity (TI000430) were obtained from Goodfellow Cambridge Ltd. Initial samples were polished by using Suba IV subpad stacked under a polytex buff pad to obtain a smooth surface finish while protecting macro scale surface contours of the implants. In addition, two different sizes of abrasive papers (silicon carbide 150C-90 µm particle size and P320- 45 µm particle size) were used in place of the polishing pad to create the microstructures through CMP. CMP slurries were prepared by using 5 wt% alumina (Al_2_O_3_) abrasives with 50 nm particle size at pH 4 using nitric acid. Slurry stability and particle size distribution was studied by using Coulter LS 13 320 laser particle size measurement instrument.

Titanium samples were polished for 2 minutes in the presence of 3 wt% H_2_O_2_ in the solution (H_2_O_2_- Sigma Aldrich, %34.5–36.5 purity) as an oxidizer to promote the chemical activity. Material removal rates were calculated through weighing the samples pre and post CMP. All samples were cleaned in ultrasonic bath by using pH adjusted DI water for 5 min and dried with nitrogen gas before they were characterized.

Fig. 1 shows the particle size distribution of the CMP slurries based on volume and number based distributions. It can be seen that the baseline slurry has a bimodal size distribution due to the agglomerates present in the slurry. The volume based size distribution measurements highlight the agglomerates better as the dominant particulate volume normalizes the data. Yet, the number based particle size distribution results show that the number percentage distribution of the agglomerates at 2-micrometer size range is negligible within the overall slurry particle population.

Before the application of CMP, original baseline titanium plates had a thick anodized oxide layer on the surface. CMP process performed in the absence of an oxidizer resulted in removal of this porous top oxide layer and exposed the bare titanium surface with a ~45 degree contact angle value, which is approximately half of the contact angle value measured on the untreated baseline sample. The remaining sample plates were CMP treated in the presence of an oxidizer in the polishing slurry. As it can be seen in Fig. 2, the change in surface roughness had a more predominant effect on the wettability, which is measured by contact angle response. Contact angle values increased as a function of surface roughness on these samples exposed to CMP (excluding the baseline sample with porous oxide on top). The highest contact angle value was obtained on the untreated baseline titanium sample, which can be attributed to the porosity of the surface oxide [2].

Fig. 2-b compares the change in the material removal rate responses by RMS surface roughness measurements obtained through AFM analyses. It can be seen that both the material removal rate and the surface roughness increases when the CMP is conducted by using abrasive papers in place of the original polytex based polishing pad material. The surface optical images also highlight the induced surface microstructures when the rough fixed abrasive polishing papers are utilized during the CMP.

Fig. 3 illustrates the surface chemical composition evaluations obtained by FT-IR analyses. The IR spectrum of both the CMP implemented and the baseline samples show a transmittance peak at ~550 cm^−1^ corresponding to the characteristic peak of Ti-O vibration in TiO_2_ [3]. The change in the peak intensity as a function of the implemented CMP treatment highlights the impact of CMP process on the surface oxide film formation. The original sample has the highest peak due to the presence of thick oxide layer. Around the 1100 cm^−1^ wavenumber, the broader peak is assigned to the Ti–O–H bond vibration on the surface [4]. The transmittance peak at ~626 cm^−1^ in the spectrum belongs to the peaks of Ti–O stretching [5]. In all the analyzed peaks, the highest intensity remains to be on the baseline sample due to its thick anodized oxide layer as compared to the nano-scale thin oxide film formation on the samples prepared by CMP implementation.

## References

[1] Ozdemir Z., Ozdemir A., Basim G.B. (2016). Biomedical applications of chemical mechanical polishing. Mater. Sci. Eng. - Part C.

[2] Campos D.M., Santos E., Kuromoto N.K., Soares G.A. (2007). Preliminary results of osteoblast adhesion on titanium anodic films. Rev. Matéria.

[3] Connor P.A., Dobson K.D., McQuillan A.J. (1999). Infrared spectroscopy of the TiO_2_/aqueous solution interface. Langmuir.

[4] Ahna K.H., Parkb Y.B., Parka D.W. (2003). Kinetic and mechanistic study on the chemical vapor deposition of titanium dioxide thin films by in situ FT-IR using TTIP. Surf. Coat. Technol..

[5] Jensena H., Solovieva A., Lib Z., Søgaarda E.G. (2005). XPS and FTIR investigation of the surface properties of different prepared titania nano-powders. Appl. Surf. Sci..

